# Advanced 2D/3D cell migration assay for faster evaluation of chemotaxis of slow-moving cells

**DOI:** 10.1371/journal.pone.0219708

**Published:** 2019-07-17

**Authors:** Lea Tomasova, Zeno Guttenberg, Bernd Hoffmann, Rudolf Merkel

**Affiliations:** 1 ibidi GmbH, Gräfelfing, Germany; 2 Institute of Complex Systems 7: Biomechanics, Forschungszentrum Jülich GmbH, Jülich, Germany; Hungarian Academy of Sciences, HUNGARY

## Abstract

Considering the essential role of chemotaxis of adherent, slow-moving cells in processes such as tumor metastasis or wound healing, a detailed understanding of the mechanisms and cues that direct migration of cells through tissues is highly desirable. The state-of-the-art chemotaxis instruments (e.g. microfluidic-based devices, bridge assays) can generate well-defined, long-term stable chemical gradients, crucial for quantitative investigation of chemotaxis in slow-moving cells. However, the majority of chemotaxis tools are designed for the purpose of an in-depth, but labor-intensive analysis of migratory behavior of single cells. This is rather inefficient for applications requiring higher experimental throughput, as it is the case of e.g. clinical examinations, chemoattractant screening or studies of the chemotaxis-related signaling pathways based on subcellular perturbations. Here, we present an advanced migration assay for accelerated and facilitated evaluation of the chemotactic response of slow-moving cells. The revised chemotaxis chamber contains a hydrogel microstructure–the migration arena, designed to enable identification of chemotactic behavior of a cell population in respect to the end-point of the experiment. At the same time, the assay in form of a microscopy slide enables direct visualization of the cells in either 2D or 3D environment, and provides a stable and linear gradient of chemoattractant. We demonstrate the correctness of the assay on the model study of HT-1080 chemotaxis in 3D and on 2D surface. Finally, we apply the migration arena chemotaxis assay to screen for a chemoattractant of primary keratinocytes, cells that play a major role in wound healing, being responsible for skin re-epithelialization and a successful wound closure. In direction of new therapeutic strategies to promote wound repair, we identified the chemotactic activity of the epithelial growth factor receptor (EGFR) ligands EGF and TGFα (transforming growth factor α).

## Introduction

Chemotaxis, cell migration prompted by an extracellular chemical gradient, is an essential biological process that underlies a wide range of physiological, as well as pathological events, for instance development, immune response, cancer metastasis, or wound repair [1–7]. Chemotactic response is directional, up (positive) or down (negative) the concentration gradient of a chemoattractant, or a chemorepellent, respectively. Intense, long-running research activities on cell migration and chemotaxis uncovered the basic mechanisms, key signaling proteins and pathways involved in this complex process [8–14]. However, most of our understanding of chemotactic response in eukaryotic cells is based on studies of *Dictyostelium discoideum* and leukocytes [15, 16], i.e. cells that migrate in an amoeboid-like manner, at a rather high speed (~ 10 μm/min), referred to as fast-moving cells. In contrast, slow-moving cells employ mesenchymal-like, adhesion-dependent migration strategy, typical for fibroblasts, cancer cells, endothelial cells or keratinocytes. Slow-moving cells migrate at a speed rate of approximately one cell-body length per hour (i.e. <1 μm/min) and their trajectories during chemotaxis are more diffusive than those of fast-moving cells, which tend to migrate almost directly towards the source of the chemoattractant [15, 17–19].

Examples of chemotaxis of both fast- and slow-moving cells can be found in the different phases of the wound healing process. The wound repair is a complex process integrating the interactions of multiple cellular components, growth factors (GFs), and extracellular matrix (ECM). The distortion of the skin barrier and the underlaying tissues by an injury initiate diverse wound-specific signals, including the release of numerous tissue and growth factors that diffuse into the surrounding areas. Within several minutes, neutrophils and phagocytes are recruited by gradients of chemokines from the circulation to the site of the wound; and once there, they move chemotactically in order to cleanse the site from pathogens and dead cells [20]. The inflammatory phase is a rapid process, established within several hours. In contrast, the next steps of wound healing, the proliferative phase, and re-epithelialization, both characterized by proliferation and migration of slow-moving cells, start within hours after the trauma, and span over several days to weeks [15, 20]. During the proliferative phase, the matrix-remodeling cells, fibroblasts, invade the temporary, fibrin-rich wound tissue, being attracted by the platelet-derived growth factor (PDGF) that is secreted by the coagulating platelets trapped in the fibrin network [21, 22]. The fibroblasts together with other proliferating cells are responsible for the formation of a novel ECM, called the granulation tissue, which fills the wound. In the process known as re-epithelialization, other slow-moving cells, the basal keratinocytes of the wound margins, migrate over the newly formed tissue of the wound bed in order to close the gap and restore the integrity of the skin barrier [23]. Keratinocyte motility is activated within several hours after the injury by multiple wound-initiated signals, including mechanical stimuli such as loss of contact inhibition at the free edges of the wound margin [24, 25], multiple tissue factors [26, 27], and growth factors that are secreted by the immune cells and fibroblasts in the wound [28–30]. Several growth factors and chemokines were described to be involved in activation of epithelial cells; however, it is yet to be understood in detail, how exactly the diverse signals control and navigate keratinocyte migration over the wound bed [29, 31]. Thus, the investigation and comprehensive understanding of the regulation mechanisms of keratinocyte motility could lead to determination of molecular targets and/or to development of new therapeutic approaches to promote wound closure.

The migration characteristics of slow-moving cells call for experiments with longer observation time (12–24 h) in order to ensure a reliable and reproducible measurement of chemotactic behavior; challenging the design of chemotaxis assays to maintain a gradient stable over long time period [32, 33]. However, the seminal instruments for studying chemotaxis were developed rather with respect to fast-moving cells, generating only temporally stable, less defined, steep gradients; e.g. pipette assay, agarose assay, or Boyden/transwell assay [1, 34–36]. Such systems furthermore omit direct observation of cell behavior and it is impossible to reliably discern between chemokinesis (general increase in migration in all directions) and directed migration (true chemotaxis) of slow-moving cells.

The need of an in-depth characterization of the chemotactic response on cellular and molecular level led to the development of chemotaxis assays for direct observation, such as Zigmond chemotaxis chamber [37] and its derivatives, Dunn [38] and Insall chamber [33], or ibidi μ-Slide Chemotaxis [32, 39] that further improved the control and longevity of the gradient. These so-called bridge assays enable direct visualization of cells seeded in an observation area bridging two reservoirs filled with solutions of different chemoattractant concentration. In the low-volume cross section of the reservoirs and the bridge, the cells are exposed to gradients that are stable over 24 hours, hence meeting the requirements of studying chemotaxis of slow-moving cells.

In recent years, the advances in micro-fabrication techniques provided for an expansion of microfluidic-based migration assays that can mimic complex 3D microenvironments and expose the cells to well-regulated external cues. The ability to fabricate precise micro-patterns from bio-compatible materials makes it possible to design elaborate, task-specific chambers that generate highly controlled gradients [1, 40–42]. However, in most of microfluidic-based chemotaxis assays, the gradient is formed by diffusion between two fluid streams, hence exposing the cells to a constant shear stress [43]. Moreover, the application of microfluidics is often challenging for non-specialist users [44].

The most severe drawback of the direct-observation chemotaxis assays is the time-demanding data analysis. Standardly, cell migration inside of the chemotaxis chamber is monitored by video-microscopy, and the evaluation of the chemotactic response is based on reconstructed migration tracks of individual cells. Real-time imaging of the cellular response provides detailed quantitative information on the whole migration process. However, it depends on specialized equipment, and is labor- and time consuming [45]. This introduces a serious obstacle, particularly for studies covering larger sample sets, typical in clinical and biomedical research.

In our study, we modified the commercially available bridge assay μ-Slide Chemotaxis with a micro-fabricated migration arena that encloses the cells to a defined area. Chemotactic response led to accumulation of cells on one side of the migration arena, an effect that could be easily recognized from the end state of the experiment. Thus, this adaptation enabled evaluation of the chemotactic effect from the end-point of the experiment alone. The end-point approach dramatically accelerated and simplified the analysis and thus substantially increased the experimental throughput. At the same time, the original features of the system were not compromised, making the assay optimal for studying chemotaxis of slow-moving cells by providing a long-term stable, convection-free linear gradient. Also, the cells were accessible for direct optical control at any time of the experiment and could be seeded in the chemotaxis chamber either on a surface (2D), or embedded in a matrix (3D).

We validated the migration arena assay by examining migration of HT-1080 fibrosarcoma cells in a gradient of fetal bovine serum (FBS), as a well-established model system for chemotaxis of slow-moving cells. Furthermore, we used the migration arena assay to study chemotaxis of normal human epidermal keratinocytes (nHEK), for the first time observing migration of primary keratinocytes in long-term stable gradients over time periods of several hours. The possibility to rapidly evaluate multiple samples with the migration arena assay enabled us to screen several growth factors for chemoattractant activity towards nHEK and to study the time course and concentration-dependency of the cellular response. Among the tested substances, transforming growth factor α (TGFα) and epithelial growth factor (EGF) were the most potent factors to induce directed migration of primary keratinocytes.

## Materials and methods

### Arena fabrication

PEG-based hydrogel barriers that frame the migration arena were fabricated directly inside a commercially available chemotaxis chamber, μ-Slide Chemotaxis (ibidi GmbH, Munich, Germany), by standard photolithography, utilizing the thiol-norbonene photopolymerization method [46, 47]. The middle channel of the chemotaxis chamber was filled with phosphate buffered saline (PBS)-based solution containing PEG monomers (2 mM 4-arm PEG4norb, Mw 20000), a crosslinker (4 mM PEG-dithiol) and a photoinitiator (3 mM lithium phenyl-2,4,6-trimethylbenzoylphosphinate; LAP). The slide was placed on a quartz photomask (purchased from Compugraphics Jena GmbH; Jena, Germany) and aligned with pattern that consisted of three duplets of rectangles (2 mm x 0.3 mm each, placed 0.4 mm apart). The spacing of the rectangle duplets on the photomask responded to the distance between the middle channels of the three chambers of the μ-Slide Chemotaxis (18.5 mm), so that the rectangles of each duplet framed one of the channels, when aligned. The polymerization was initiated by a 10 s exposure to UV light (365 nm; 10 mW cm^-2^ intensity; LED lamp KSL70/365 was purchased from RappOpto Electronic GmbH, Hamburg, Germany). The non-polymerized material was rinsed away with PBS. The chemotaxis chambers with the hydrogel arenas were stored filled with PBS at 4°C. Before experiments, chambers were equilibrated by overnight incubation at 37° C.

### Gradient characterization

In order to visualize the chemical gradient in the migration arena, the arena was filled with collagen type I matrix (1.5 mg/ml), and the diffusion of fluorescent molecules through the matrix was observed. To establish the gradient, one of the reservoirs was filled with PBS and the other with a fluorophore solution. 1 μM Alexa Fluor488 or 0.1 mg/ml FITC-tagged dextran, 40 kDa (both diluted in PBS) were used in order to represent diffusion of molecules of different sizes. The fluorescence gradient was detected by time-lapse imaging of the gradient region of the migration arena by an inverted microscope (Nikon Eclipse Ti, Nikon GmbH, Düsseldorf, Germany) equipped with a motorized stage (TI-SH-W) and FITC filter set (Nikon), 60x oil-objective (CFI PLAN Apochromat, Nikon), and CCD camera ORCA-Flash 4.0-LT (Hamamatsu Photonics, Hamamatsu City, Japan). The microscope was controlled by the Micro-Manager software [48]. Two rows of nine images across the middle part of the chemotaxis chamber, covering the 400 μm wide arena, were taken every 30 minutes, with a step of 50 μm between the frames. The center of the rows was aligned with the center of the arena. The Z-plane was focused in the middle of the collagen layer, approximately 35 μm above the slide surface. Additional micrographs were taken in the fluorophore-containing reservoir, at the extension of each row. Fluorescence intensity in a 10 μm^2^ area in the center of each frame was measured using the NIH (National Institute of Health) ImageJ software [49, 50]. The measured signal was normalized to the maximal signal of the fluorophore taken in the reservoir at each time point. Graphs show the normalized mean signal of the two rows. Control measurement *c*_*0*_ (background signal control) was taken in the same chamber before adding the fluorophore to the reservoirs. Positive control *c*_*100*_ represents the fluorescent signal measured in a chamber containing 1 μM Alexa Fluor488, or 0.1 mg/ml FITC-tagged dextran in both reservoirs, as well as in the 3D collagen matrix in the arena.

### Cell culture

HT-1080 fibrosarcoma cells (DSMZ, Braunschweig, Germany) were cultured in high glucose Dulbecco’s Modified Eagle’s Medium (DMEM) supplemented with 10% fetal bovine serum (FBS; both Sigma-Aldrich, St. Louis, MO, USA). Normal human epithelial keratinocytes (nHEK; CellSystems, Troisdorf, Germany) were cultured in DermaLife basal medium supplemented with DermaLife K LifeFactors kit (both Lifeline Cell Technology, Frederick, MD, USA), and Penicillin/Streptomycin antibiotics (Gibco, Waltham, MA, USA). The final component concentrations in the supplemented (complete) DermaLife medium were 5 μg/ml insulin, 6 mM L-glutamine, 1 μM epinephrine, 5 μg/ml apo-transferrin, 100 ng/ml hydrocortisone hemisuccinate, 0.4% bovine pituitary extract, 100 U/ml Penicillin, and 100 μg/ml Streptomycin. Only nHEK cells up to passage number 5 were used. The cultures were maintained at 37° C and in 5% CO_2_ humidified atmosphere.

### Cell viability assay

The viability of cells in the migration arena was determined by differential live and dead staining with fluorescein diacetate (FDA) and propidium iodide (PI). HT-1080 embedded in 3D collagen matrix at 10^6^ cells/ml were seeded in the migration arena, or in the standard μ-Slide Chemotaxis (for control). The reservoirs of the chemotaxis chamber were filled with medium containing 0% or 10% FBS and the chambers were incubated at 37° C and 5% CO_2_. Fluorescent micrographs of the cells were taken initially, and after 24 and 48 hours. The number of all viable and dead cells (stained green and red, respectively) in the migration arena was determined using the Cell Counter plugin for the NIH software ImageJ. Approximately 200 cells were counted per arena. Cell viability was quantified as the percentage of the viable cells in the arena.

### Chemotaxis assays

#### μ-Slide Chemotaxis assay

A chemotaxis experiment in the standard μ-Slide Chemotaxis (without migration arena) was performed according to the protocol provided by the manufacturer, as described previously [51]. For 2D experiments, the narrow channel was first coated with 1 mg/ml fibronectin for 1 hour at 37° C, before seeding 2×10^6^ cells/ml HT-1080. For a 3D experiment, HT-1080 cells were mixed with neutralized solution of bovine collagen type I (PureCol). To yield a final concentration of 10^6^ cells/ml and 1.5 mg/ml collagen, 150 μl collagen I (3 mg/ml) was mixed with 20 μl 10xDMEM, 6 μl 1M NaOH, 14 μl deionized H_2_O, 10 μl 7.5% NaHCO_3_ and 50 μl 1xDMEM (for control samples with uniform 10% FBS, 30 μl of 1xDMEM was replaced by FBS), and 50 μl of 6x concentrated cell suspension was added to the collagen solution. Cell-containing solution was loaded into the chemotaxis channel; and after cell adhesion or collagen gelation, the reservoirs of the chemotaxis chamber were filled with DMEM medium containing 0% or 10% FBS. A phase-contrast video of cell migration at a 4x magnification was recorded for 24 hours, with 10 minutes time-lapse interval on an Olympus CKX41 inverted microscope (Olympus Scientific Solutions, Waltham, MA, USA) equipped with a motoric stage (MW Tango, Märzhäuser Wetzlar GmbH, Wetzlar, Germany), 4x UPlan FLN objective (Olympus), and an on stage incubation system (ibidi GmbH, Munich, Germany). Trajectories of 35–45 cells in the observation area of each image sequence were tracked manually using the NIH ImageJ Manual Tracking plugin. The chemotactic effect was evaluated using the ibidi Chemotaxis and Migration Tool, according to the manufacturer’s instructions. Several values characterizing cell migration and chemotaxis are computed from the trajectories by the software, such as forward migration indices (FMI), velocity, and directness. All parameters generated by the software are described in detail in [39]. Briefly, FMI express the directionality of migration (i.e., the efficiency of a cell to migrate in direction of a given chemotactic stimuli), and are computed as follows:

$$FMI=\frac{1}{n}\sum_{i=1}^{n}\frac{y_{i,end}}{d_{i,accum}}$$

where *n* denotes number of cells, *y*_*i*, *end*_ coordinates of cell end point, and *d*_*i*, *accum*_ the length of the trajectory travelled by cell. Directness expresses the straightness of the cell path, irrespective to the gradient direction, and is calculated as the ratio of the Euclidian (straight-line) distance (*d*_*i*, *euclid*_), and accumulated distance travelled by the cell:

$$d_{euclid}=\sqrt{{\left(x_{i,end}-x_{i,start}\right)}^{2}+{\left(y_{i,end}-y_{i,start}\right)}^{2}}$$

$$D=\frac{1}{n}\sum_{i=1}^{n}\frac{d_{i,euclid}}{d_{i,accum}}$$

The velocity was computed as the ratio of the accumulated distance of the cell path, and the time of migration.

#### Migration arena chemotaxis assay

Before experiments, PBS was carefully removed from the chemotaxis chamber and the migration arena was coated with fibronectin (1 mg/ml in PBS, 1 hour at 37° C) for 2D experiments, or with collagen I (0.3 mg/ml PureCol in purified water for 1 hour at 37° C) for 3D experiments. Afterward, the migration arena was rinsed with PBS. Cells were trypsinized, re-suspended in complete culture media or in collagen solution at a cell concentration of 1–2.5×10^6^ cells/ml, and seeded into the migration arena by rinsing 2×10 μl of the cell suspension through the arena channel. To avoid trapping air-bubbles in the channel, the channel volume was not aspirated between the rinsing steps. After cell adhesion (1–2 h) or collagen gelation (1 h), the reservoirs were filled with media containing the respective concentrations of chemoattractant. All filling ports of the chemotaxis chambers were sealed with plugs, and cells in the arena were imaged with phase-contrast microscopy at 4x magnification for 24 hours. Spatial positions of all cells in the migration arena were determined initially and at the end-point of the experiment. The chemotactic effect was then computed as the displacement of the average spatial position of all cells in the gradient direction (center of mass displacement in gradient direction; COMD).

In a control experiment with inhibited cell proliferation, the medium in the arena channel was replaced after the cells attached with a complete medium containing 10 μg/ml mitomycin C (MMC). The cells were treated with MMC for 3 hours at 37 °C. Then, the channel was washed two times with MMC-free medium, before filling the reservoirs with the chemoattractant solutions. In the experiments with EGFR (epithelial growth factor receptor) inhibitors AG-1478 and EGFR antibody 225, the inhibitors were simply added in the medium (both in the channels and the reservoirs) when the gradient was applied, i.e. with the chemoattractant solutions.

### Statistical analysis

If not indicated otherwise, experiments were performed in at least three independent replicates, and data are presented as mean ± standard error (SEM). To test for statistical differences, one-way, or two-way ANOVA followed by Dunnett’s multiple comparison test was performed, considering p = 0.05 as the level of significance. Graphs and statistical analysis were made in the GraphPad Prism 7 software (GraphPad Software, USA). Significantly different results are indicated in the graphs with stars (* p<0.05; ** p<0.01; *** p<0.001; **** p<0.0001).

### Materials

The hydrogel monomers, 4-arm PEG4norb, were purchased from JenKem Technology USA (Plano, TX, USA). The photoinitiator LAP was synthetized as described previously [52]. The fluorescent dye Alexa Fluor488 was obtained from Invitrogen (Waltham, MA, USA), and propidium iodide from Carl Roth GmbH (Karlsruhe, Germany). Trypsinized bovine collagen type I, PureCol, was obtained from Advanced BioMatrix (Carlsbad, CA, USA), and fibronectin from Corning GmbH (Wiesbaden, Germany). Purified water was filtered with MilliQ system, Merck Millipore, Burlington, MA, USA. TGFα, insulin and BPE (bovine pituitary extract) were supplied as parts of the DermaLife K LifeFactors kit from Lifeline Cell Technology (Frederick, MD, USA). Human recombinant EGF (*E*. *coli*-derived) was obtained from PromoCell (Heidelberg, Germany), and recombinant human TGFβ-1 (CHO-derived) was purchased from Peprotech (Hamburg, Germany). EGF receptor monoclonal antibody 225 was obtained from Life Technologies (Carlsbad, CA, USA). All other reagents; i.e., PEG-dithiol; FITC-dextran, 40 kDa; 10xDMEM; 7.5% NaHCO_3_; fluorescein diacetate; mitomycin C; and EGFR inhibitor AG-1478, were obtained from Sigma-Aldrich (St. Louis, MO, USA).

## Results and discussion

### Migration arena chemotaxis assay development

**Fig 1:**
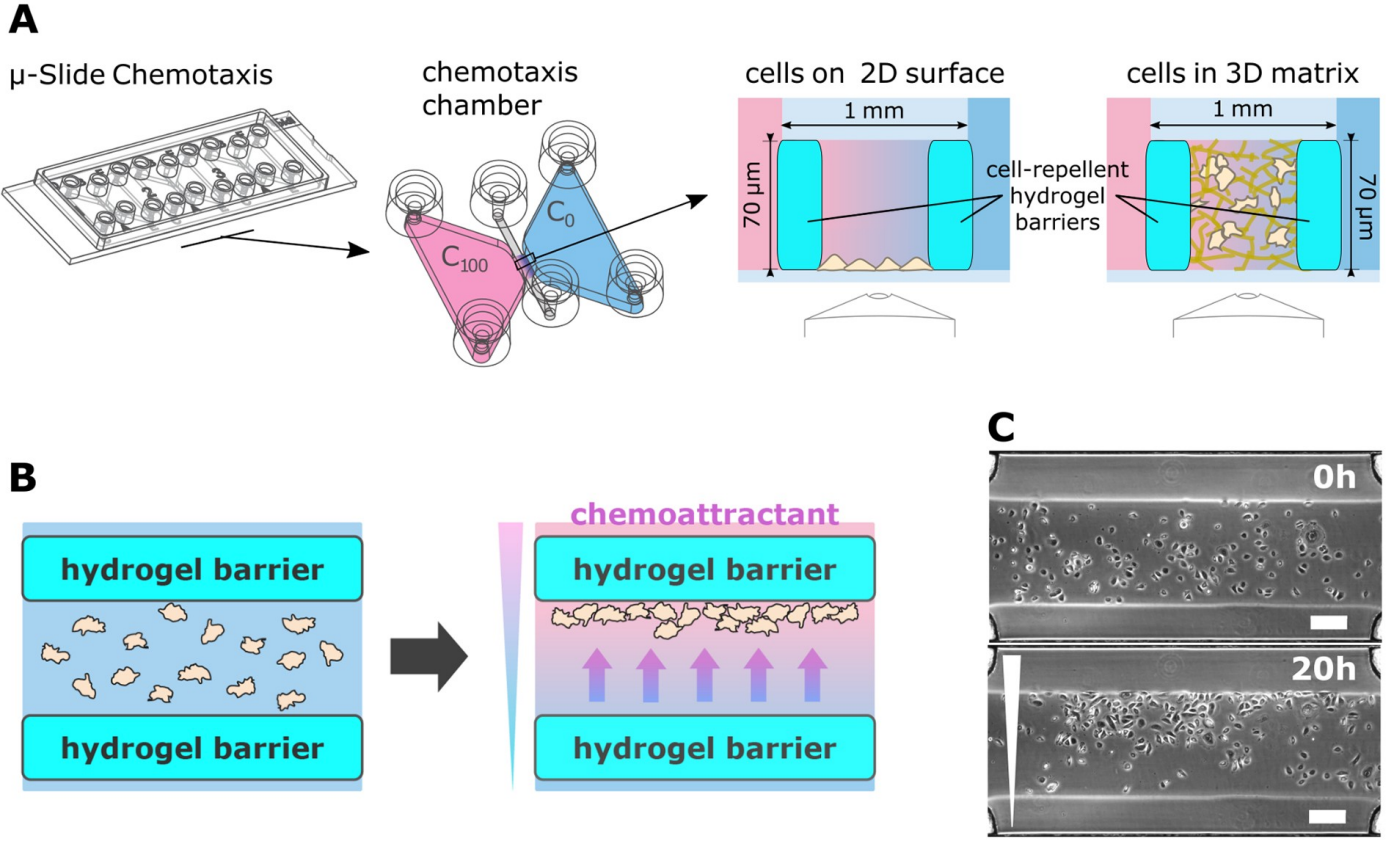
μ-Slide Chemotaxis with a migration arena. **A.** μ-Slide Chemotaxis was supplemented with a hydrogel migration arena, positioned in the gradient region of the chemotaxis chamber. Dimensions and volumes are indicated or given in the text. **B.** Function of the migration arena. Cell migration was restricted to the area of the migration arena by barriers from cell-repellent hydrogel. Initially, cells were distributed uniformly in the migration arena. When a gradient was established across the arena, cells migrated in the direction of the chemoattractant and accumulated at one side of the arena. Thus, chemotaxis could be identified from the end-point micrograph. **C.** Example of keratinocytes distribution in the migration arena at the beginning of the experiment and after 20 hours of migration in a gradient of TGFα. The micrographs are representative examples of the sample set evaluated in Fig 4. Scale bar = 200 μm.

The migration arena assay is based on the μ-Slide Chemotaxis, a microfluidic tool with the size of a microscopic slide (Fig 1A). The slide carries three independent chemotaxis chambers. Each chamber consists of two symmetrical reservoirs of 65 μl volume apiece, bridged by a narrow channel of 140 nl. Both reservoirs and the channel have two filling ports that can be independently sealed with specially designed plugs. Cells are seeded in the narrow middle channel (in 2D or embedded in a 3D matrix) and the reservoirs are filled with medium containing different concentrations of chemoattractant. A stable and linear gradient is formed across the cell-containing bridge area [51]. Since the cells are distributed homogeneously in the observation area, and they are able to migrate freely in and out from the gradient area to the reservoirs, it is not possible to detect a chemotactic effect solely from the distribution of the cells at the end-time of the experiment. Instead, here, a preferred direction of cell migration can be identified only by time-lapse imaging followed by the time-demanding analysis of the complete cell trajectories.

In our modification of the assay, we equipped the chemotaxis chamber with hydrogel barriers to separate the middle channel from the reservoirs, thus restricting cell migration to the defined area of the migration arena (Fig 1A, right). The small mesh size (tens of nanometers; [53]) of the PEG-based non-adhesive hydrogel prevented cells from migrating into the reservoirs, yet did not hinder diffusion of chemoattractants and gradient formation. In case of a chemotactic effect, the cells migrated towards the increasing concentration of the chemoattractant and accumulated on one side of the arena. This set-up enabled fast evaluation of the chemotactic behavior with respect to the end-point of the chemotactic behavior, based on the change in the cell distribution (Fig 1B and 1C).

We optimized the chemistry and geometry of the micro-structures so that stable barriers were formed, enclosing a migration arena wide enough to accommodate a sufficient number of cells for chemotaxis analysis. The rectangular barriers were placed on the border of the channel and the reservoir, each 300 μm wide, 2 mm long (along the lenght of the cross-section of the channel and the reservoirs), and filling the height of the channel, i.e. 70 μm. Thus, the inner width of the migration arena was 400 μm. Fabrication of wider arenas that could hold more cells was problematic, since thin barriers (<200 μm) were unstable and prone to rupture. The mechanical stability of the arena was also dependent on the stiffness of the hydrogel, which correlates with the polymer concentration and the ratio of the polymer and the crosslinker in the gel [54]. On the other hand, too concentrated polymer solution (<2 mM PEG4norb) in the chemotaxis channel inclined to unspecific polymerization of the hydrogel that could block the channel between the barriers.

Before the experiments, the migration arena surface had to be coated with an adhesive protein. For 2D experiments cell adhesion had to be enabled. Here, the arena was coated with fibronectin to ensure optimal attachment and migration of the cells. For 3D experiments with cells embedded in 3D collagen, the stability of the collagen matrix in the rather thin channel between the hydrogel barriers had to be improved. In these experiments, the arena was pre-coated with solubilized collagen that anchored the collagen matrix to the bottom of the slide and prevented its shrinking.

#### Characterization of the concentration gradient in the migration arena

**Fig 2:**
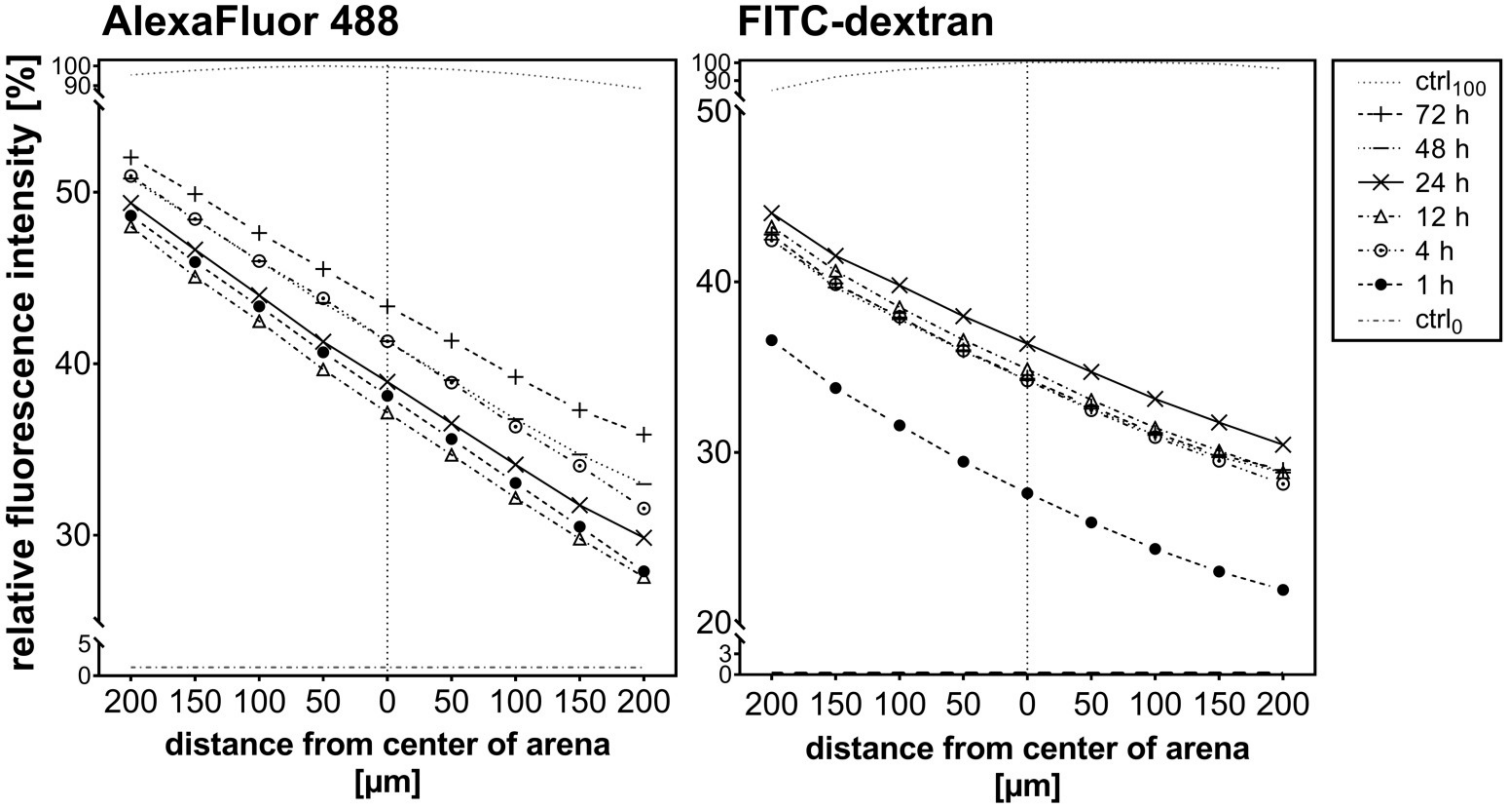
Visualization of the gradient in the migration arena. Gradients remained stable over 72 hours in the migration arena. The fluorescence signals of Alexa Fluor 488 (643 Da; left) and FITC-dextran (40 kDa; right) were measured across the arena to represent the gradients of small and large molecules, respectively. Control measurements were performed in absence of fluorophore (ctrl_0_), and in a chamber uniformly filled with the maximal concentration of fluorophore (ctrl_100_). Graphs show representative results of at least three experiments; origin of the x-axis represents the middle of the migration arena.

Stability and shape of the gradient formed in the migration arena were verified by fluorescent microscopy. Signaling compounds that are present in the cell environment vary in size. Motogenic growth factors are typically small polypeptides with a molecular weight below 30 kDa [55, 56]. Here, we used AlexaFluor 488 dye (643 Da) and 40 kDa FITC-labelled dextran (FITC-dextran) to represent the diffusion of molecules of different sizes (the largest growth factor applied in our experiments was TGFβ-1, which has a 24 kDa dimeric precursor form). The results shown in Fig 2 indicated that a linear gradient was formed across the cell-containing region of the migration arena, and remained stable over the whole 72 hours observation period. Diffusion and gradient formation of FITC-dextran was slower in comparison to AlexaFluor 488; however, by 4 hours after start of the experiment, the gradient of the larger molecule also reached a stable linear state.

#### Evaluation of chemotactic effect in migration arena assay

Chemotaxis in the migration arena was evaluated by comparing the cell distribution at the beginning and at the end of the chemotaxis experiment. It was quantified by the displacement of the center of mass in the direction of the concentration gradient—COMD. The center of mass is defined here as the spatial average of all cell positions. Only the component along the gradient (arbitrarily assigned the y-direction) was considered. Coordinates of all *n* cells in the arena were determined from micrographs taken initially (y_i, start_), and at the end (y_i, end_) of the experiment. The y-axis originates from the edge of the arena. Center of mass displacement (COMD) was then computed as the difference of the average cell ordinate at the start and at the end-point of the experiment:

$$COMD={COM}_{end}-{COM}_{start}=\left[\frac{1}{n}\sum_{i=1}^{n}\left(y_{i,end}\right)\right]-\left[\frac{1}{n}\sum_{i=1}^{n}\left(y_{i,start}\right)\right]$$

If the cells migrate randomly in all directions, the cell distribution in the arena does not change over time—COM_end_ is similar to COM_start_, resulting in COMD ≈ 0. In contrast, accumulation of the cell mass at one side of the arena as result of directed migration produces COMD ≠ 0. A value of COMD that exceeds the standard deviation expected for *n* cells, randomly distributed in the migration arena, indicates chemotactic behavior.

We used the NIH ImageJ software and the command ‘Analyze particles’ to determine cell ordinates. The software automatically recognized individual cell bodies on a binary image and returned the spatial position of each cell. However, poor image quality, high cell density or complex matrix (as 3D collagen fibers) thwarted automatic cell recognition in some cases. There, manual analysis was employed (by using the ImageJ plugin Cell Counter). Though the manual analysis was more labor intensive, it was still much faster than the manual cell tracking through long movies that is usually required to reconstruct cell trajectories in standard chemotaxis assays. The migration arena normally contained 100–200 cells, and the cell position was determined on two micrographs (*start* and *end*); therefore, manual determination of each cell position required approximately 400 mouse-clicks per sample. For comparison, migration of HT-1080 cells in the standard μ-Slide Chemotaxis assay is typically recorded for 24 hours with a 10 minutes time-lapse interval [32, 39]; therefore, manual tracking analysis would require to determine the position of each cell 144 times. To obtain statistically relevant results, trajectories of at least 30–40 cells are needed, resulting in more than 4000 mouse-clicks. Usually, the analysis of one sequence by manual tracking takes about 30–45 minutes, while the end-point analysis is done manually within minutes, and automated within seconds.

If suitable, the end-point analysis can be further simplified by employing fluorescent microscopy instead of phase-contrast imaging—e.g. by fluorescent labelling of cell nuclei for a more stable automatic cell detection. Furthermore; if the initial cell distribution in the arena is reliably homogenous, COM_start_ can be considered constant and the chemotactic effect determined solely from the end-point cell distribution (COMD = COM_end_). In our experiments, the COM_start_ was stably superimposed with the center of the arena, with a standard deviation within 3% of the arena width. It should be noted however, that the uniformity of cell seeding can vary with respect to the cell type, cell density, and type and quality of substrate coating; and an asymmetrical initial cell distribution in the arena could bias the results, if neglected in the analysis. Therefore, the seeding uniformity should be verified for every experiment.

### HT-1080 chemotaxis towards fetal bovine serum in 2D and 3D

**Fig 3:**
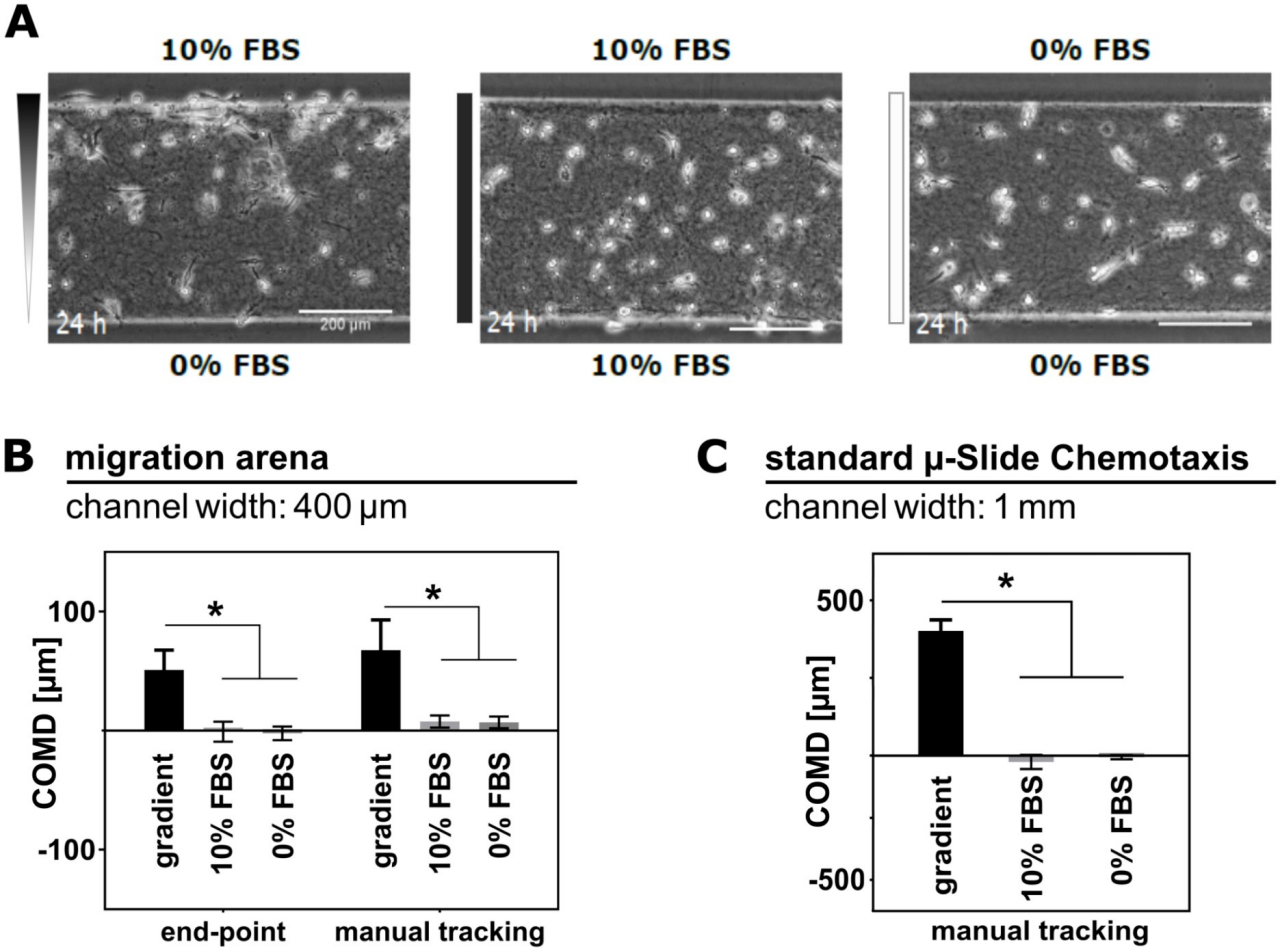
Evaluation of HT-1080 chemotaxis in 3D collagen. **A.** Migration of HT-1080 in 3D collagen in migration arenas was recorded for 24 hours in FBS gradient (0–10%), or in uniform FBS concentration (10% and 0%). **B.** Mean COMD was determined by end-point, and manual tracking analysis. For the end-point analysis, positions of all cells (100–150) in the arena were determined initially and after 24 hours. By manual tracking trajectories of 35–40 cells in each arena were reconstructed. **C.** For a comparison, the experiment was repeated in the standard μ-Slide Chemotaxis. All bar graphs show mean COMD ± SEM (n = 3); * indicate significantly different means (ANOVA analysis followed by Dunnett’s test; p<0.05).

Directed migration of fibrosarcoma cells in a chemical gradient of FBS is a well-established model of chemotaxis of slow-moving cells [39]. HT-1080 typically migrate at a speed rate of one cell-body length in one hour (ca. 50 μm/h), employing mesenchymal, anchorage-dependent type of migration [57, 58]. To validate the arena assay, we analyzed chemotaxis of HT-1080 in a migration arena both in 2D and in 3D environment, and compared the results with data obtained in the standard μ-Slide Chemotaxis assay. As control, the directional response towards 10% FBS was compared to random, un-directional migration of HT-1080 in uniform environment of medium containing either 0% or 10% FBS (in 3D: Fig 3A; the results of HT-1080 chemotaxis in 2D environment are shown in Supporting Information, S1 Fig). Time-lapse videos of HT-1080 migrating in the arenas were recorded for 24 hours, with a time-lapse interval of 10 minutes. First and last image of the sequence were analyzed applying the end-point approach. As shown in the graph in Fig 3B, COMD in FBS gradient (51 μm) is significantly higher than COMD in uniform environment (<2 μm), indicating the expected chemotactic effect. The result of the end-point analysis was verified by manual tracking of 30–40 cells in each arena through the whole recorded sequence (Fig 3B, right). From the trajectories, we could also compute additional parameters of the cell migration in the arena, as forward migration indices (FMI, [39, 51]), velocity, or directness (S2 Fig). The experiment was repeated under the same conditions using the standard μ-Slide Chemotaxis assay, and the chemotactic behavior was analyzed by manual tracking (Fig 3C). The results of both assays show an explicit trend correctly identifying chemotactic behavior of HT-1080 in a gradient of FBS, which is not present in the control experiments with uniform environment. It should be noted that the COMD values generated by the end point analysis describe the integral behavior of all cells in the migration arena, whereas the manual tracking reflects the migration of a randomly selected portion of the cell population (30–40 cells). Only complete migration tracks can be included in the manual tracking analysis; therefore, dead and immotile cells, as well as cells that are lost and reappear again in the observation field are not considered. In contrast, in the end-point analysis, all cells of the population contribute to COMD value. Besides, the absolute values of COMD in the migration arena and in the standard chemotaxis slide differ due to the distinct width of the respective cell-containing area (1 mm in the standard chamber, and 400 μm in the arena), and the absence of confinement in the standard chemotaxis chamber. Therefore, quantitative comparison between the absolute values of COMD retrieved by the different analytical methods and tools is not possible.

In the arena, the distance travelled was limited by the hydrogel barriers, and with increasing time also by the cell mass accumulating along the barrier. Once a cell reached the barrier, it stalled; whereas in the standard chamber, cells could leave the gradient region, and indeed migrated into the reservoirs. For similar reasons, trajectories of cells in the arenas were deformed along the direction of barriers (x-axis). Cells that were migrating towards the chemoattractant and reached the hydrogel barrier normally underwent one of the following scenarios: 1) arrest of migration; the cell stayed close to the barrier; 2) change of direction; migration back towards the center until chemotactic stimuli prevailed and the cell turned back toward the barrier; 3) attempt to persist in forward migration, resulting in sliding movement along the hydrogel barrier (see S1 and S2 Videos; ESI). Nevertheless, for the purpose of correctly identifying chemotactic behavior, migration perpendicular to the gradient could be safely ignored.

The standard assay allowing unconfined cell migration in all directions is an optimal approach for studying migration of single cells, generating parameters that are computed from complete trajectories of individual cells (such as directness or velocity). However, we have verified that for correct identification and reliable quantification of chemotaxis, the laborious analysis based on manual tracking is not required, and can be replaced by the arena assay and the much faster end-point analysis.

### Chemotaxis of keratinocytes

One of the physiological processes where migration of slow-migrating cells plays a crucial role is skin reepithelization. In 1988, Martinet et al. [59] observed that keratinocyte migration is stimulated by fibroblasts and smooth muscle cells conditioned media, and rat wound fluid. By present days, a motogenic effect of several distinct GFs and chemokines on these cells was identified, as reviewed in [31, 60, 61]. However, by now, keratinocyte migration has only been studied in experiments where the chemical agent was applied either non-directionally, or in a non-specified, non-linear and short-term gradient (i.e., Boyden/transwell assay [59, 62–66]). Here, we exposed primary keratinocytes for the first time to long-term stable and spatially well-defined gradients of GFs, and analyzed the migratory response quantitatively in order to identify optimal conditions that stimulate directional migration of these cells. We have selected five agents that were previously reported to affect keratinocyte migration: EGF, TGFβ-1, TGFα, insulin, and BPE (bovine pituitary extract) [63, 66–71].

**Fig 4:**
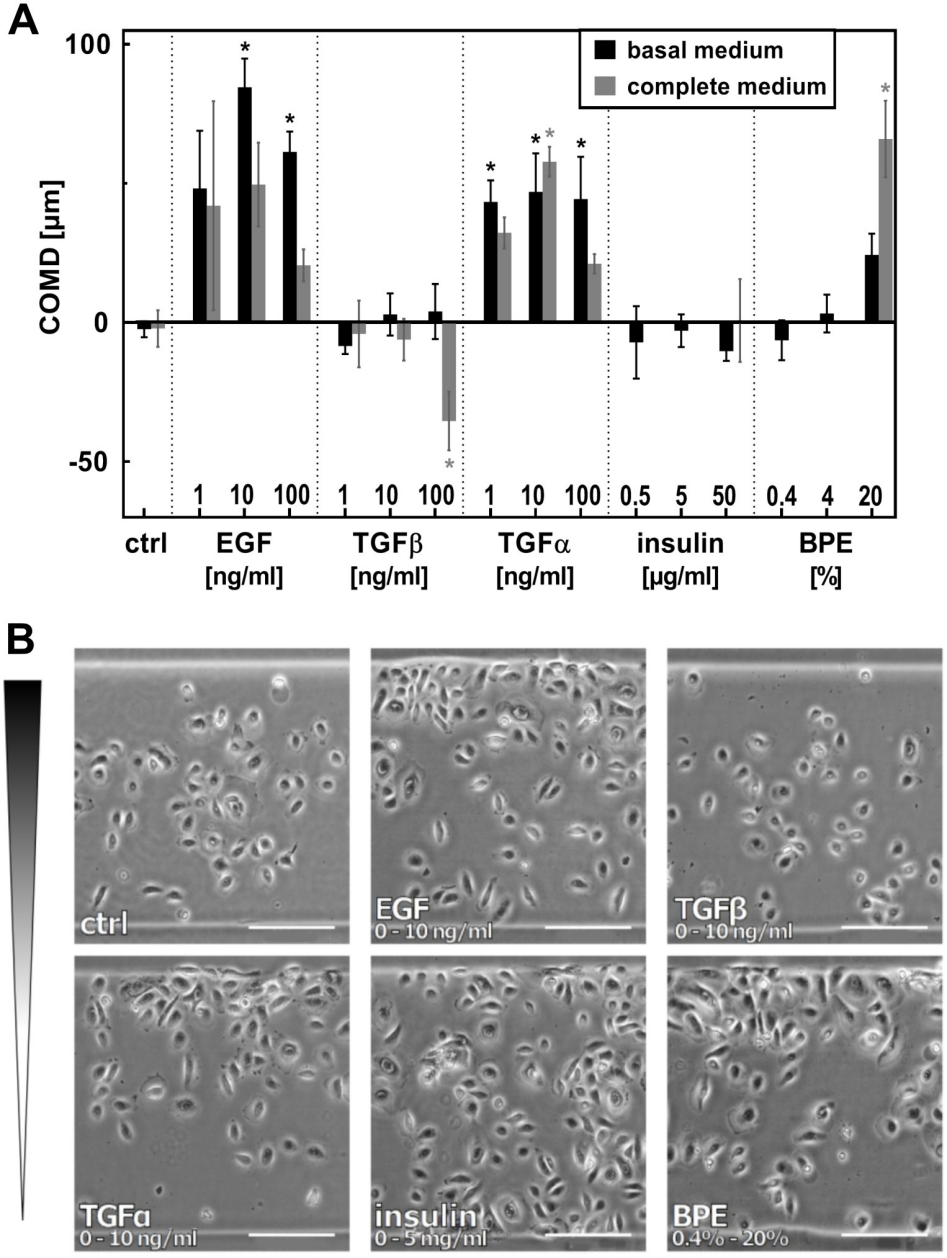
EGF, TGFα and BPE induce directed migration of nHEK. **A.** nHEK cells were seeded in migration arenas coated with fibronectin. Gradients of EGF, TGFα, TGFβ, insulin, and BPE were established in the chemotaxis chamber, and the effect on cell migration was evaluated after 20 hours. The maximal concentration of each gradient is indicated in the graph. All gradients start from zero, with the exception of insulin and BPE, which were contained already at low concentrations in complete medium (0.4% BPE, 5 μg/ml insulin). In basal medium, the gradients of these factors also started from zero. Mean COMD ± SEM (n = 4); * indicate means significantly different from control (ANOVA followed by Dunnett’s test; p<0.05). Mean COMD ± SEM is also listed in a table in S5 Fig. **B.** Micrographs show cell distribution in the arenas at the end-point of the experiment (scale bars = 200 μm).

Keratinocytes migrate over the wound along the basal membrane–i.e., in 2D-like conditions. Therefore, the cells were seeded in fibronectin-coated arenas in 2D, and the reservoirs were filled with blank background medium or solution of the chemotactic agent, respectively. For each chemical agent, three gradients of increasing steepness were tested, using either basal (BM) or complete (CM) keratinocyte culture medium as background solution (Fig 4A). Complete medium, standardly used for cultivation of nHEKs, contains bovine pituitary extract; i.e. a source of unspecified mixture of growth factors, that might affect the migratory behavior of cells in an unspecific manner. Previous studies with other cell types showed that using a defined, serum-free medium could increase the sensitivity of chemotaxis assays [51]. Therefore, we were interested whether a similar effect is observed in the chemotactic behavior of nHEK cells in the migration arena assay. First, we determined the optimal time span of the experiment. Migration in gradients was recorded for 24 hours with 1 hour time-lapse interval. COMD of all 24 frames of the sequence was computed by automated cell recognition (S4 Fig). If chemotaxis was observed, COMD peaked 10–15 hours after start of the experiment, and kept stable from there on. Therefore, we selected 20 hours as an optimal end-point time of our analysis.

Micrographs in Fig 4B show the cell distribution in the arena after 20 hours migration in selected gradients. The results clearly indicated chemotactic activity of EGF and TGFα. The nHEK cells were also attracted to 20% BPE in CM; however, BPE in lower concentrations did not induce directed migration. TGFβ and insulin exhibited no chemotactic activity, apart from an unexpected negative peak of 100 ng/ml TGFβ in CM. We verified by reconstructing the cell trajectories that this accumulation of cells in the distant part of the migration arena (relative to the TGFβ source) indeed resulted from directed migration in the opposite direction of the gradient (S7 Fig). There are conflicting pieces of evidence on the effect of TGFβ signaling in epithelial cells, in respect to the character of the cellular response, spanning from terminal differentiation to increase in motility (reviewed in [72, 73]). However, to our knowledge there is no data that would explain negative chemotaxis in response to TGFβ.

**Fig 5:**
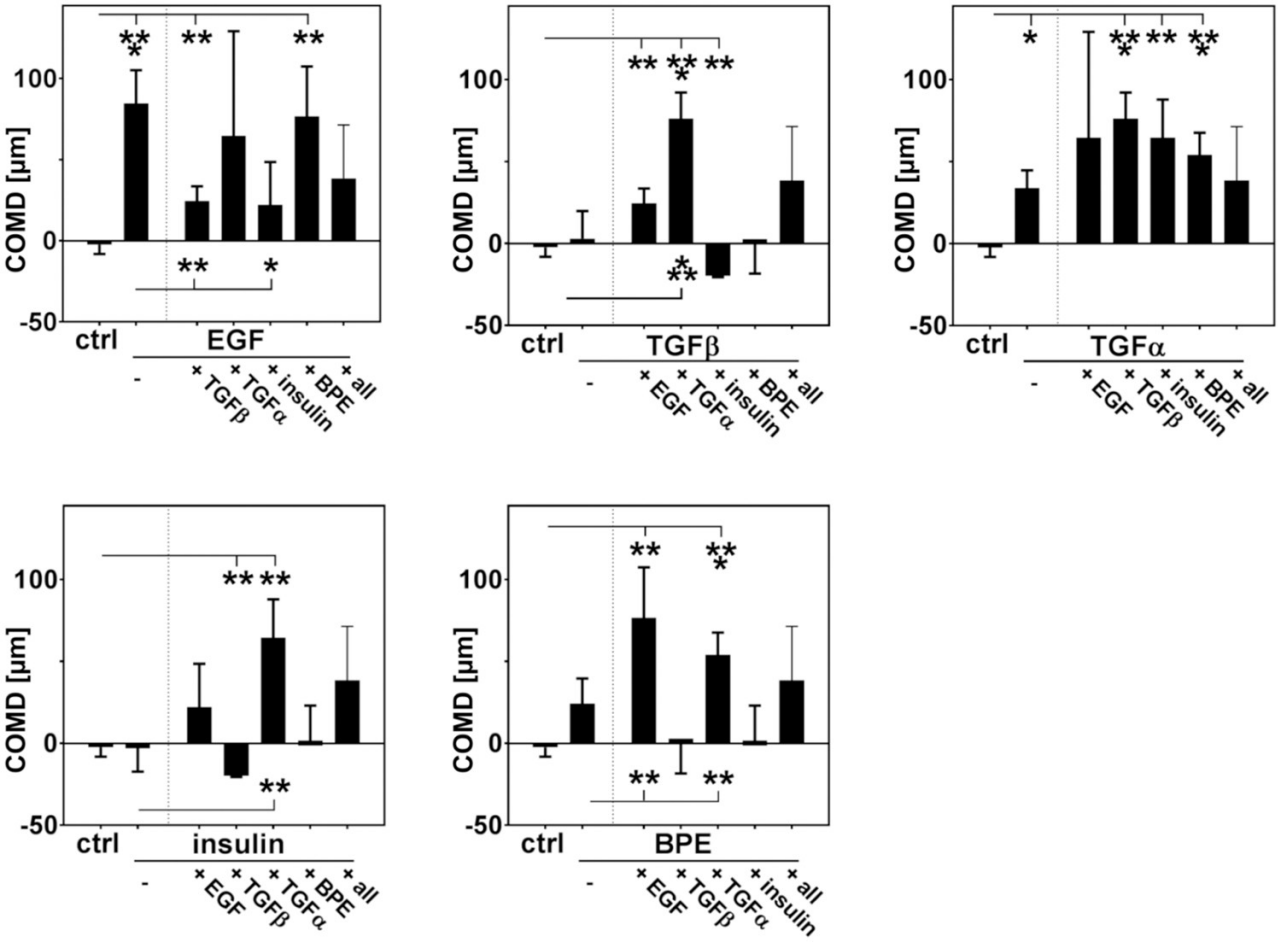
Effect of combined growth factors on nHEK chemotaxis. Chemotaxis of nHEK in gradients of combined motogenes in basal medium was evaluated after 20 hours of migration. Experiments were performed in basal medium, maximal concentrations of the GFs gradients were 10 ng/ml EGF, TGFα and TGFβ, 5 μg/ml insulin, 4% BPE; the concentration range of all gradients starting from zero. The combined substances were applied in gradients of the same direction. ‘All’ represents gradient of solution containing all GFs. Mean COMD ± SEM (n = 4); * indicate means significantly different from control (ANOVA followed by Dunnett’s test; p<0.05).

Control experiments showed no significant difference between COMD of normally proliferating cells and cells treated with a proliferation inhibitor (S6 Fig), verifying that the observed changes of cell distribution in the migration arena resulted from chemotaxis, i.e. directed migration, and not from increased GF-stimulated cell growth close to the barrier. From these results we concluded that the different outcomes in basal and complete medium (compare COMD in BM and CM; Fig 4A) did not arise from different growth rates in the different media. Instead, our results suggest the presence of some factors in the medium that interfered with the chemotactic effect, e.g. BPE or insulin. We also tested the chemotactic activity of two and more GFs applied simultaneously (Fig 5). In those experiments we observed that insulin significantly decreased EGF-stimulated chemotaxis. Thus, this chemical that is also present in CM might indeed have such an interfering effect. Previously, insulin has been reported as a potent promotor of keratinocyte migration and proliferation [31, 69, 74]. It is possible, that increased random migration (chemokinesis) caused by insulin interfered with directed motion in the arena assay. Chemotactic response to EGF was also reduced by TGFβ. Surprisingly, while neither TGFβ nor insulin showed chemotactic activity in BM on itself, a gradient of both of these factors mixed together resulted in a negative chemotactic effect, similar as observed by TGFβ gradient in CM. On the other hand, the chemotactic activity of TGFα, which induced chemotaxis in both media (Fig 4A), was stable and unaffected by addition of other GFs. All together, these results demonstrated that executing chemotaxis experiments in basal medium, not supplemented with serum or GF-containing additives, can improve the sensitivity of the assay.

#### EGF- and TGFα-induced chemotaxis of nHEK is EGFR-dependent

**Fig 6:**
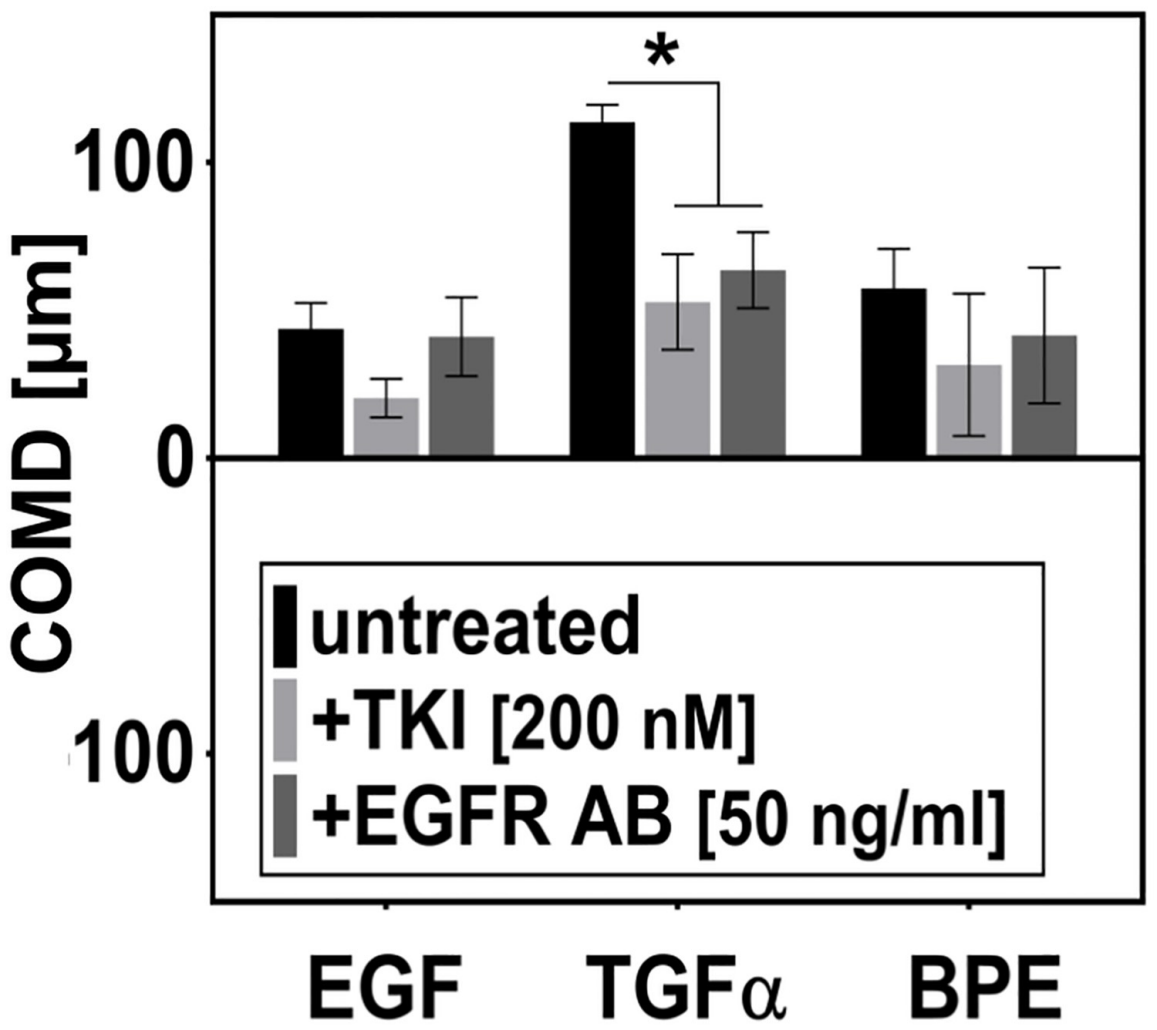
EGF- and TGFα-induced chemotaxis of nHEK is EGFR-dependent. Signals from both EGF and TGFα are transferred into the cell by EGFR. Cells chemotaxing in gradients of EGF (0–10 ng/ml, in BM), TGFα (0–10 ng/ml, in BM), and BPE (0.4%-20%, in CM) were treated by EGFR inhibitors (EGFR antibody, EGFR AB, 50 ng/ml; and EGFR tyrosine kinase inhibitor tyrphostin AG-1478, TKI, 200 nM). COMD was evaluated after 20 hours. Mean COMD ± SEM (n = 4); * indicate means significantly different from control (ANOVA followed by Dunnett’s test; p<0.05).

The structurally similar molecules TGFα and EGF are both ligands of epithelial growth factor receptor (EGFR) [75–77]. Ligand binding leads to dimerization of the receptor and activation of its intracellular tyrosine-kinase (TK) activity. Signal transduction pathways triggered by EGFR are responsible for coordinating several important cellular processes, including cell proliferation and cell motility [78]. In order to verify the specificity of the detected chemotaxis response to EGF and TGFα, we studied GF-induced chemotaxis in presence of EGFR inhibitors (Fig 6). Inhibiting the EGFR tyrosine receptor kinase with the intracellular inhibitor (tyrphostin AG-1478) led to a decrease in EGF- and TGFα-stimulated chemotaxis. In contrast, blocking ligand binding to the receptor by an EGFR-specific antibody only affected chemotactic response to TGFα. These different outcomes argue for binding of EGF and TGFα to the receptor with a different affinity, and functional selectivity of the ligands, which was already described before [79–81]. We also detected a chemotactic activity of BPE, which is an unspecified mix of growth factors, presumably containing also EGFR ligands. Therefore, we hypothesized that BPE-induced chemotaxis could be also at least partially mediated by EGFR. However, while there seems to be a similar trend in the results when compared to EGF-induced chemotaxis, the differences in COMD were not statistically significant.

## Conclusions

Most state-of-the-art chemotaxis assays designed for slowly moving cells concentrate on comprehensive low-throughput analysis of cell migratory behavior. However, for some scientific questions a high experimental throughput may be more important than depth of detail. Unfortunately, chemotactic tools developed specifically for high throughput applications were primarily designed for fast-moving cells, typically neutrophils [82–85]. The presented migration arena chemotaxis assay is a suitable platform for studying slow cell migration in both 2D and 3D. In contrast to other end-point chemotaxis assays, it provides a flow-free linear gradient that remains stable for more than 48 hours. The defined gradient, as well as the possibility of direct imaging of the cell distribution in the symmetrical chemotaxis chamber at the end of the experiment make it possible to distinguish between chemokinesis, and genuine chemotaxis, i.e. directional migratory response induced by the chemoattractant. By replacing the lengthy real-time analysis of single cell trajectories required by the standard bridge chemotaxis assays with the evaluation based on the end-point of the experiment, the migration arena increases the experimental throughput substantially. For illustration, for this manuscript we evaluated two experimental data sets, one on HT-1080 chemotaxis in 2D and 3D, and the other on nHEK migration in gradients of GFs. HT-1080 chemotaxis set consisted of six samples in three biological replicates each. Due to the complex morphology of HT-1080 cells and collagen background in the 3D experiments, the initial and end-point cell positions were analyzed manually. The end-point analysis of such dataset could be easily processed in less than two hours. Similar dataset was generated by the standard chemotaxis assay in a control experiment. Typically, manual tracking of 6–8 time-lapse sequences is manageable in one working day; thus, the analysis of this dataset took 2–3 days, with approximately 12 hours of continuous tracking. Within the nHEK experiments, we evaluated more than 60 different samples in 3–4 biological replicates. Using the automated end-point analysis, the data could be evaluated in several days; therefore, the limiting factor for the study duration is the time-scale of the experimental procedure. Data analysis of similar datasets by tracking through time-lapse videos would take several months. The end-point approach therefore considerably facilitates experiments that handle a large number of samples, such as screenings for chemoattractants, as well as for micro-environmental cues (protein coating, etc.), genetic mutations, or inhibitors that affect directed migration of slow-moving cells. Such experiments can help to comprehend and mimic a specific microenvironment and external cues that distinct cell types sense and respond to, and consequently to develop therapeutics that would affect chemotactic cell behavior in case of pathology (e.g., neutralizing tumor cell invasion by inhibiting the ability of the cells to sense chemical gradients; impairing tumor vascularization; or accelerating skin reepithelization by activating skin cells and luring them into the wounded area.) However, end-point migration tools are often mistrusted, since they evaluate the migratory behavior of the cell population as a whole, and usually do not provide a possibility to control the state of the cells during the experiment [37, 44, 86]. In any cell population, a variability in the migratory response has to be taken in account, caused for example by dead or immotile cells. If the fraction of such cells is very high, the results of the end-point experiment could be considerably biased. The migration arena, though, enables optical control at any time of the experimental procedure, as the cells are seeded in an observation area accessible for inverted microscopes. Therefore, the homogeneity of the cell population can be easily verified, and it is also possible to record cell migration in the arena for follow-up detailed analysis of chemotactic behavior, as illustrated in S2 and S7 Figs. The end-point approach can be with benefit applied to identify substances with chemotactic potency from a large set of candidates, and select those of interest for a more detailed analysis of migratory behavior on single-cell level by manual tracking.

We demonstrated some of the possible applications of the migration arena while studying chemotaxis of primary keratinocytes. Although these cells employ the mesenchymal strategy of migration and their response to extracellular stimuli is not immediate, chemotaxis tools that are not optimal for slow-moving cells, such as the Boyden chamber/transwell assay, have been widely applied to study migration of these cells [59, 62–64, 66, 87]. Results of these experiments can clarify which GFs affect keratinocyte migration; however, since it is not possible to correctly recognize proper chemotaxis (i.e., a directional migratory response towards the GF) from chemokinesis (increased migration in random direction) in this type of migration assays, no conclusion can be drawn on the chemotactic potency of the studied GFs based on their results. Employing the migration arena assay, we were able to evaluate the chemotactic activity in a range of chemical conditions. From the tested GFs, EGFR ligands EGF and TGFα were the strongest chemoattractants of nHEK cells. EGFR signaling is crucial for effective reepithelization, and inhibition of EGFR kinase activity *in vivo* leads to significant delay in wound healing [88, 89]. It is therefore not surprising that its ligands are potent chemokinetic factors of epithelial cells [62, 66, 71]. Our results show that the cell migratory response to these factors *in vitro* is indeed directional; thus, GFs of the EGF family could efficiently navigate keratinocytes by inducing chemotaxis, in absence of other directional chemical or mechanical cues. Furthermore, a quantitative analysis of the chemotactic effect enabled us to optimize the experimental parameters to yield the best-readable response, and thus establish a chemotaxis model that can be further utilized to study the mechanisms of GF-induced directed migration of primary keratinocytes and similar cells.

## Supporting information

S1 Fig Evaluation of HT-1080 chemotaxis in 2D. **A.** Migration of HT-1080 in 2D in fibronectin-coated migration arenas was recorded for 24 hours in a FBS gradient (0–10%), or in uniform FBS concentration (10% and 0%). **B.** Mean COMD determined by end-point and manual tracking analysis. For the end-point analysis, the positions of all cells (100–150) in the arena were determined initially, and after 24 hours. By manual tracking, trajectories of 35–40 cells in each arena were reconstructed. Only complete trajectories of cells can be included in the statistical analysis. Therefore, the manual tracking statistics is biased by the selection of cells that are alive and motile during the whole time of the experiment. **C.** For comparison, the experiment was repeated in the standard μ-Slide Chemotaxis. All bar graphs show mean COMD ± SEM (n = 3); * indicate significantly different means (ANOVA analysis followed by Dunnett’s test; p<0.05).
(TIF)

S2 Fig Migration trajectories of HT-1080 in 3D and 2D arena. Trajectories of cells migrating in migration arena in 3D **(A)** and 2D **(B)** were reconstructed by manual tracking, and analyzed with the Migration and Chemotaxis software. Forward migration indices (FMI), velocity and directness were computed. FMI express the efficiency of migration toward the chemoattractant and are computed as the ratio of the distance travelled by the cell in the gradient direction, and the complete (accumulated) length of the travelled path. All bar graphs show mean COMD ± SEM (n = 3); * indicate significantly different means (ANOVA analysis followed by Dunnett’s test; p<0.05). Red crosses in trajectories plots indicate COMD of the end-points of the tracks.
(TIF)

S3 Fig Cell viability is not affected in the migration arenas. **A.** HT-1080 embedded in 3D collagen were cultivated in migration arenas or standard μ-Slide Chemotaxis (ctrl), in gradient or constant concentration of FBS. The viability was evaluated by live/dead staining with fluorescein diacetate (FDA) and propidium iodide (PI). Bars represent mean rate of viable cells in the arenas + SD (n = 3). The viability in arenas is not significantly different from the control (ANOVA analysis). **B.** Live/dead staining of HT-1080 cells in migration arena in gradient of 10% FBS (concentration increases upwards). Cells are stained with FDA (viable cells, green) and PI (dead, red). In average, 200 cells were counted per arena. Scale bar = 100 μm.
(TIF)

S4 Fig Time-lapse analysis of nHEK chemotaxis. Time-lapse videos of nHEK cells migrating in fibronectin coated arenas in gradients of several motogenes in basal (BM; black bars) or complete medium (CM; grey bars) were recorded for 24 hours with an 1 hour time-lapse interval. COMD was determined by end-point analysis after each hour in order to select the time of best response. Bar graphs show mean COMD ± SEM (n = 4) determined by the analysis of cell positions in each frame; all graphs are scaled identically. Maximal concentrations of gradients are stated in the graphs. Data were analyzed with ANOVA test followed by Dunnett’s multiple comparisons test (t_0_ vs. t_n_); * indicate means significantly different from t_0_.
(TIF)

S5 Fig GF-stimulated chemotaxis of nHEK cells. COMD [μm] of nHEK cells migrating for 20 hours in gradients of GFs in basal (BM) and complete medium (CM) are listed in the table. Data are as well presented in the form of graph in Fig 4A.
(TIF)

S6 Fig Proliferation control. Cell proliferation was inhibited with mitomycin C (MMC) in a control chemotaxis experiment in order to verify that the uneven cell distribution in migration arena is caused by directed migration (true chemotaxis), and is not dependent on cell growth. In order to probe whether increased proliferation of cells in complete medium masked chemotaxis, we used MMC on those samples that gave different results in basal and complete medium (gradients of EGF, BPE). However, no significant differences between MMC-treated and normally proliferating cells were found. Bars show mean COMD ± SEM (4 arenas were analyzed for each condition; each arena contained 150–200 cells). COMD of MMC-treated and untreated cells was compared with multiple t-test; p<0.05).
(TIF)

S7 Fig Chemorepellent effect of TGFβ. Experiments on nHEK cells (Fig 4) showed a surprising negative chemotaxis effect of a 0–100 ng/ml TGFβ gradient in complete medium. In order to verify that the accumulation of the cells at the distant barrier of the migration arena (in respect to the highest TGFβ concentration) was indeed caused by a chemorepellent effect, we analyzed cell trajectories by manual tracking in the time-lapse sequences acquired during this experiment. The hairplot graph shows the complete trajectories of cells that migrated in the migration arena for 24 hours in the gradient of TGFβ (0–100 ng/ml in complete medium). Cell migration was recorded with time-lapse microscopy with an interval of 10 min, and the trajectories of 35–40 randomly selected cells in each arena were reconstructed by manual tracking. The trajectories were analyzed with the Chemotaxis and migration tool software. Values of chemotactic parameters, such as forward migration indices along the gradient direction (FMI_y_ = -0.09 ± 0.05 [a.u.]; n = 3), or center of mass displacement along the gradient direction (COM_y_ = -73 μm ± 18 μm; n = 3) indicate negative chemotaxis. Shown is a representative result of three independent experiments. The direction of the TGFβ gradient is indicated by the arrow. The end-points of the tracked cells are indicated in the plot by the black dots, and the red cross represents the center of mass displacement. The evaluated raw data were acquired in the same experiment that was shown in Fig 4 and S5 Fig.
(TIF)

S1 Video Chemotaxis of nHEK cells in a gradient of TGFα in the migration arena. nHEK cells were seeded in a fibronectin coated arena, and a gradient of TGFα was established. The concentration of TGFα increased from 0 ng/ml to 10 ng/ml in the direction indicated by the arrow. Cell migration was recorded for 24 hours with a time-lapse interval of 10 minutes. Scale bar = 200 μm.
(MP4)

S2 Video Chemotaxis of HT-1080 in 3D matrix in the migration arena. HT-1080 cells embedded in 3D collagen I matrix migrated in a gradient of FBS. The concentration of FBS increased from zero to 10% in the direction indicated by the arrow. Cell migration was recorded for 24 hours with a time-lapse interval of 10 minutes. Scale bar = 100 μm.
(AVI)
