# Supplementary figures and images for: Advanced 2D/3D cell migration assay for faster evaluation of chemotaxis of slow-moving cells
Source: PLoS One. 2019 Jul 17;14(7):e0219708. doi: 10.1371/journal.pone.0219708 (PMC6636736; doi:10.1371/journal.pone.0219708)

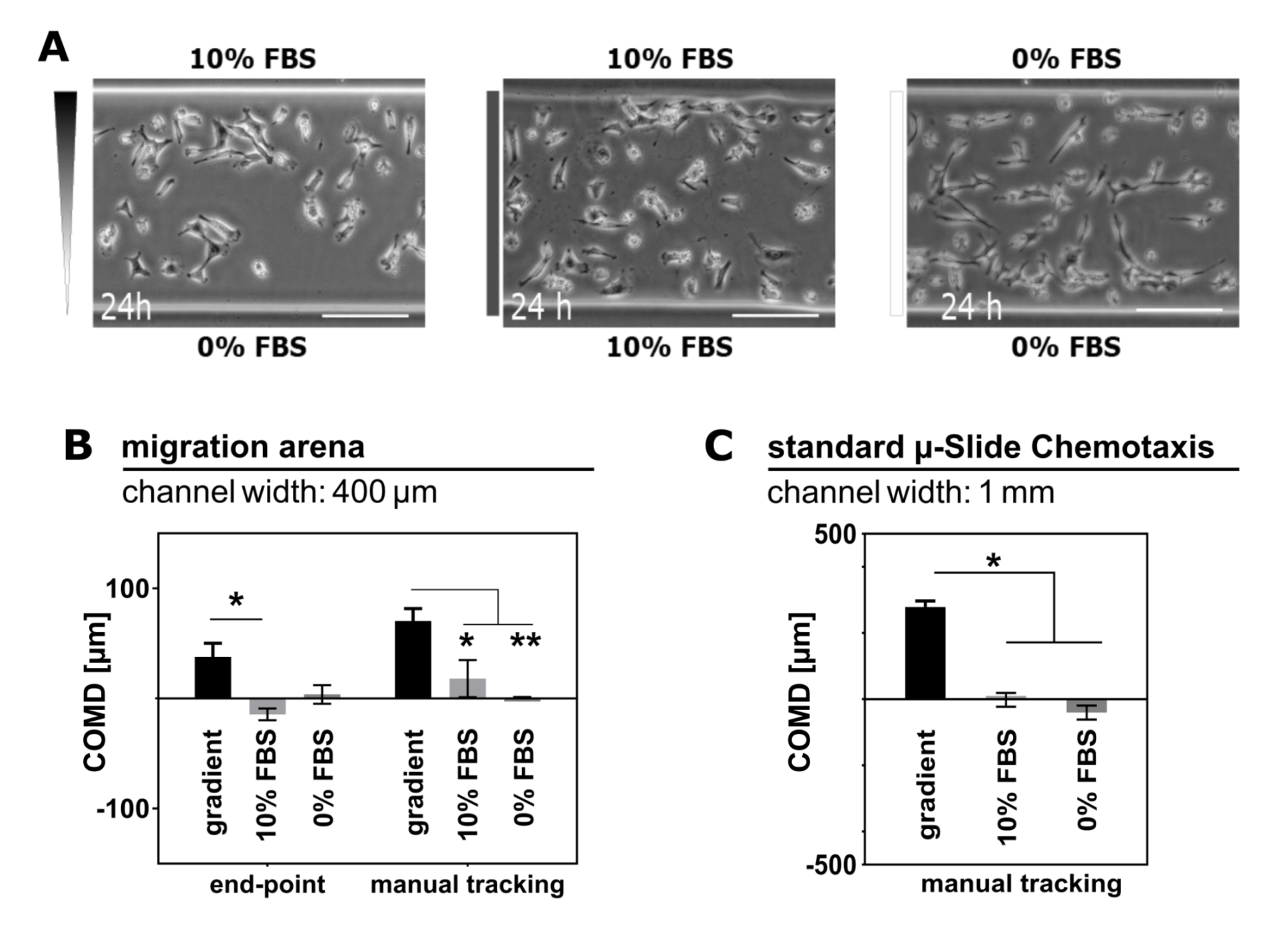

Supplement: S1 Fig — A. Migration of HT-1080 in 2D in fibronectin-coated migration arenas was recorded for 24 hours in a FBS gradient (0–10%), or in uniform FBS concentration (10% and 0%). B. Mean COMD determined by end-point and manual tracking analysis. For the end-point analysis, the positions of all cells (100–150) in the arena were determined initially, and after 24 hours. By manual tracking, trajectories of 35–40 cells in each arena were reconstructed. Only complete trajectories of cells can be included in the statistical analysis. Therefore, the manual tracking statistics is biased by the selection of cells that are alive and motile during the whole time of the experiment. C. For comparison, the experiment was repeated in the standard μ-Slide Chemotaxis. All bar graphs show mean COMD ± SEM (n = 3); * indicate significantly different means (ANOVA analysis followed by Dunnett’s test; p<0.05). (TIF) [file pone.0219708.s001.tif]

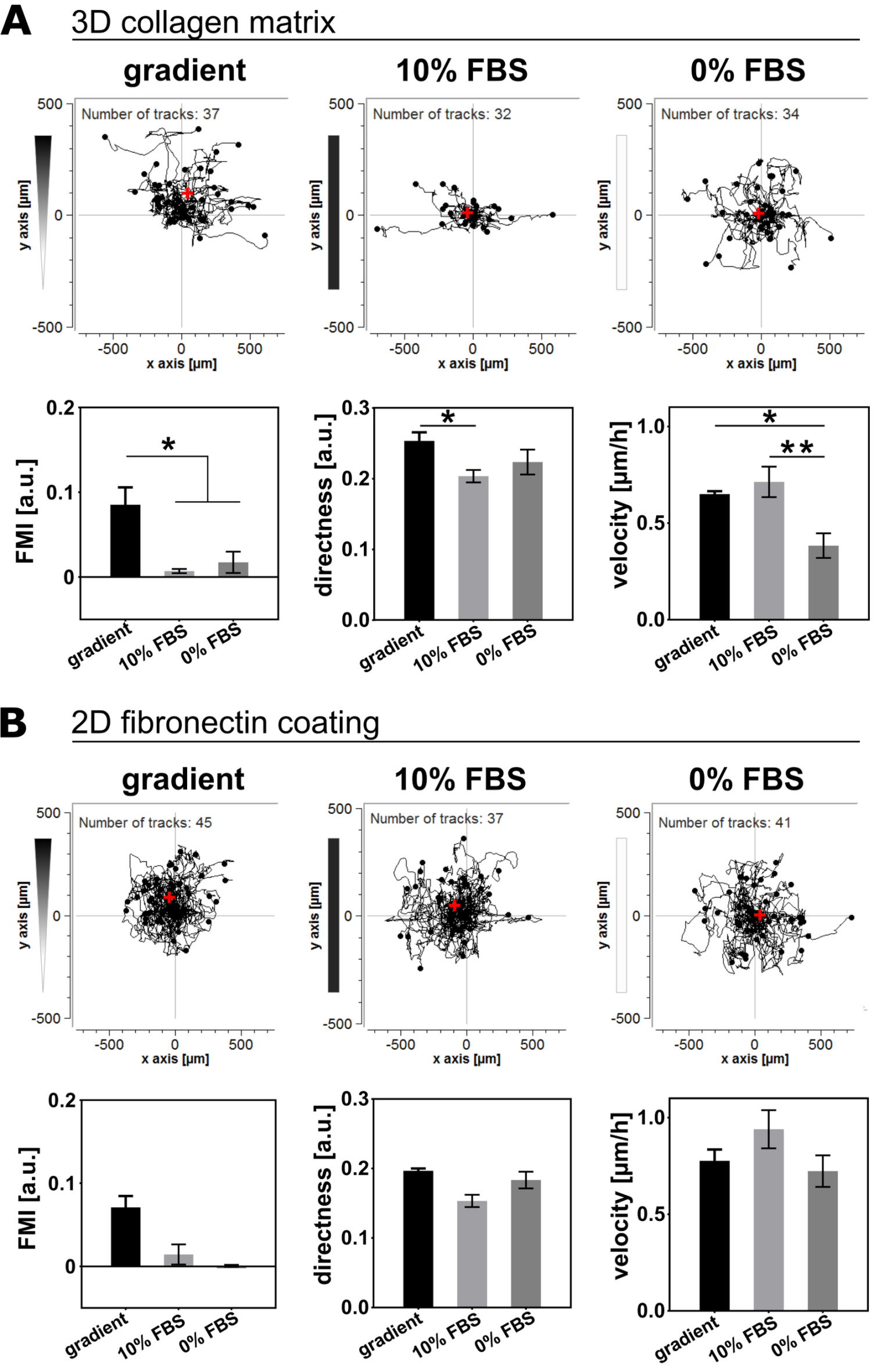

Supplement: S2 Fig — Trajectories of cells migrating in migration arena in 3D (A) and 2D (B) were reconstructed by manual tracking, and analyzed with the Migration and Chemotaxis software. Forward migration indices (FMI), velocity and directness were computed. FMI express the efficiency of migration toward the chemoattractant and are computed as the ratio of the distance travelled by the cell in the gradient direction, and the complete (accumulated) length of the travelled path. All bar graphs show mean COMD ± SEM (n = 3); * indicate significantly different means (ANOVA analysis followed by Dunnett’s test; p<0.05). Red crosses in trajectories plots indicate COMD of the end-points of the tracks. (TIF) [file pone.0219708.s002.tif]

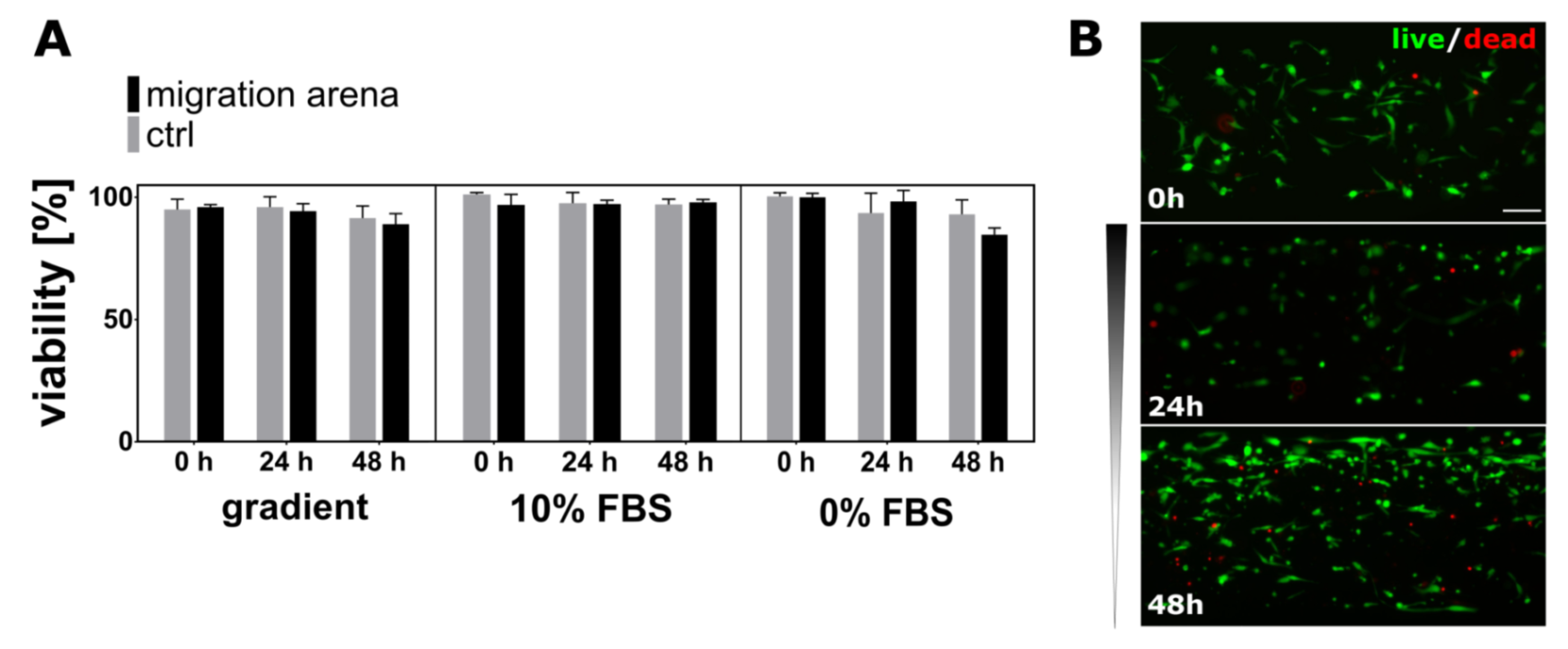

Supplement: S3 Fig — A. HT-1080 embedded in 3D collagen were cultivated in migration arenas or standard μ-Slide Chemotaxis (ctrl), in gradient or constant concentration of FBS. The viability was evaluated by live/dead staining with fluorescein diacetate (FDA) and propidium iodide (PI). Bars represent mean rate of viable cells in the arenas + SD (n = 3). The viability in arenas is not significantly different from the control (ANOVA analysis). B. Live/dead staining of HT-1080 cells in migration arena in gradient of 10% FBS (concentration increases upwards). Cells are stained with FDA (viable cells, green) and PI (dead, red). In average, 200 cells were counted per arena. Scale bar = 100 μm. (TIF) [file pone.0219708.s003.tif]

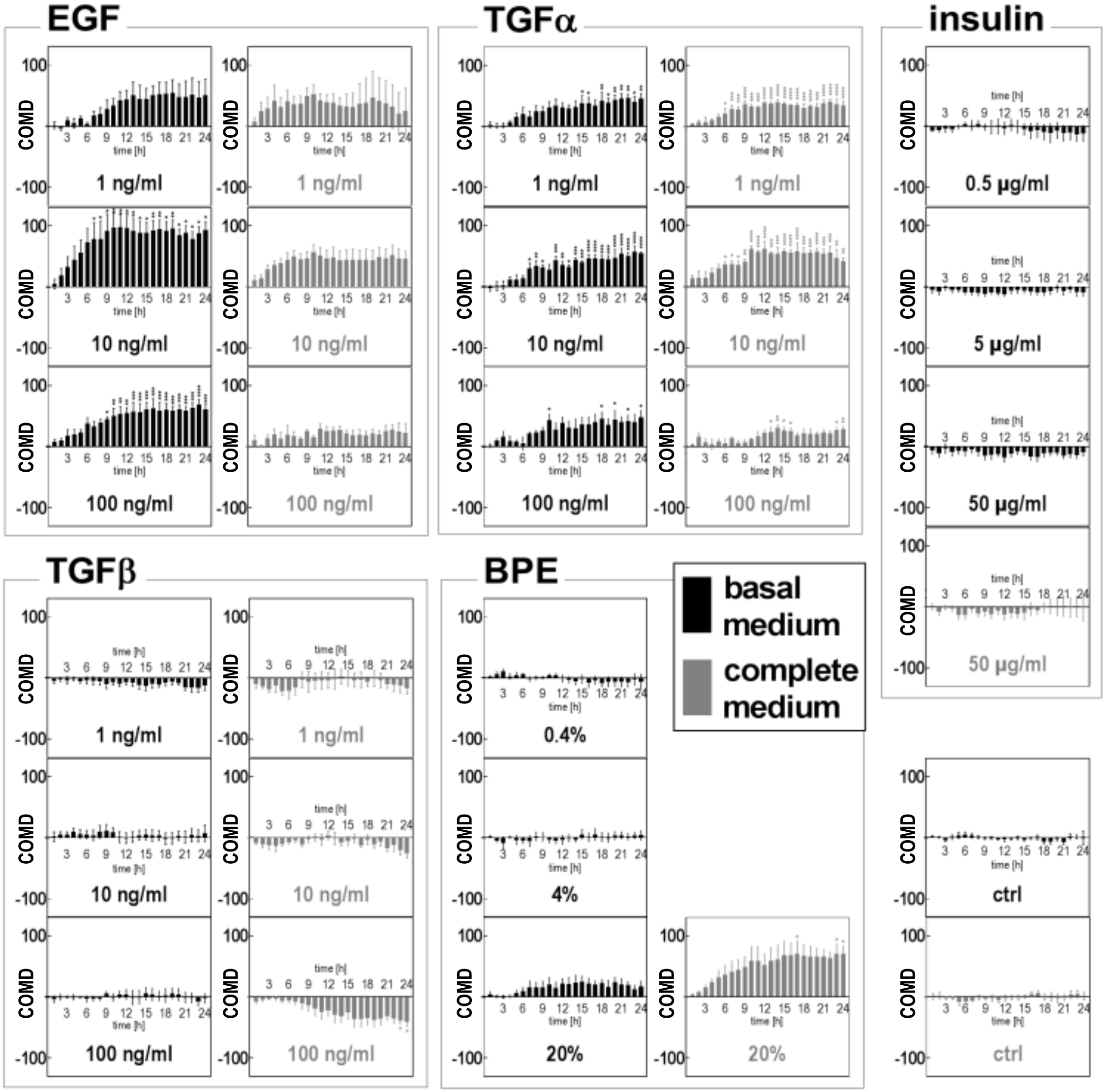

Supplement: S4 Fig — Time-lapse videos of nHEK cells migrating in fibronectin coated arenas in gradients of several motogenes in basal (BM; black bars) or complete medium (CM; grey bars) were recorded for 24 hours with an 1 hour time-lapse interval. COMD was determined by end-point analysis after each hour in order to select the time of best response. Bar graphs show mean COMD ± SEM (n = 4) determined by the analysis of cell positions in each frame; all graphs are scaled identically. Maximal concentrations of gradients are stated in the graphs. Data were analyzed with ANOVA test followed by Dunnett’s multiple comparisons test (t0 vs. tn); * indicate means significantly different from t0. (TIF) [file pone.0219708.s004.tif]

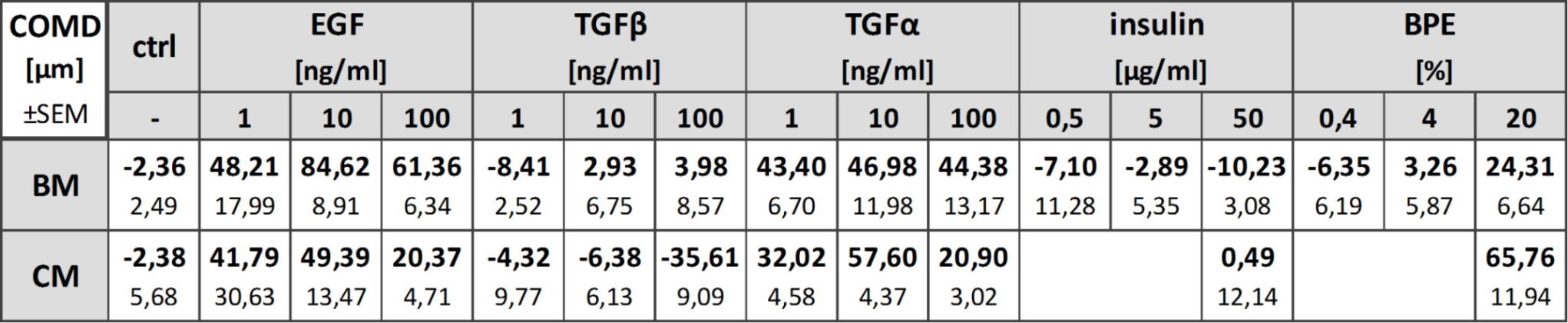

Supplement: S5 Fig — COMD [μm] of nHEK cells migrating for 20 hours in gradients of GFs in basal (BM) and complete medium (CM) are listed in the table. Data are as well presented in the form of graph in Fig 4A. (TIF) [file pone.0219708.s005.tif]

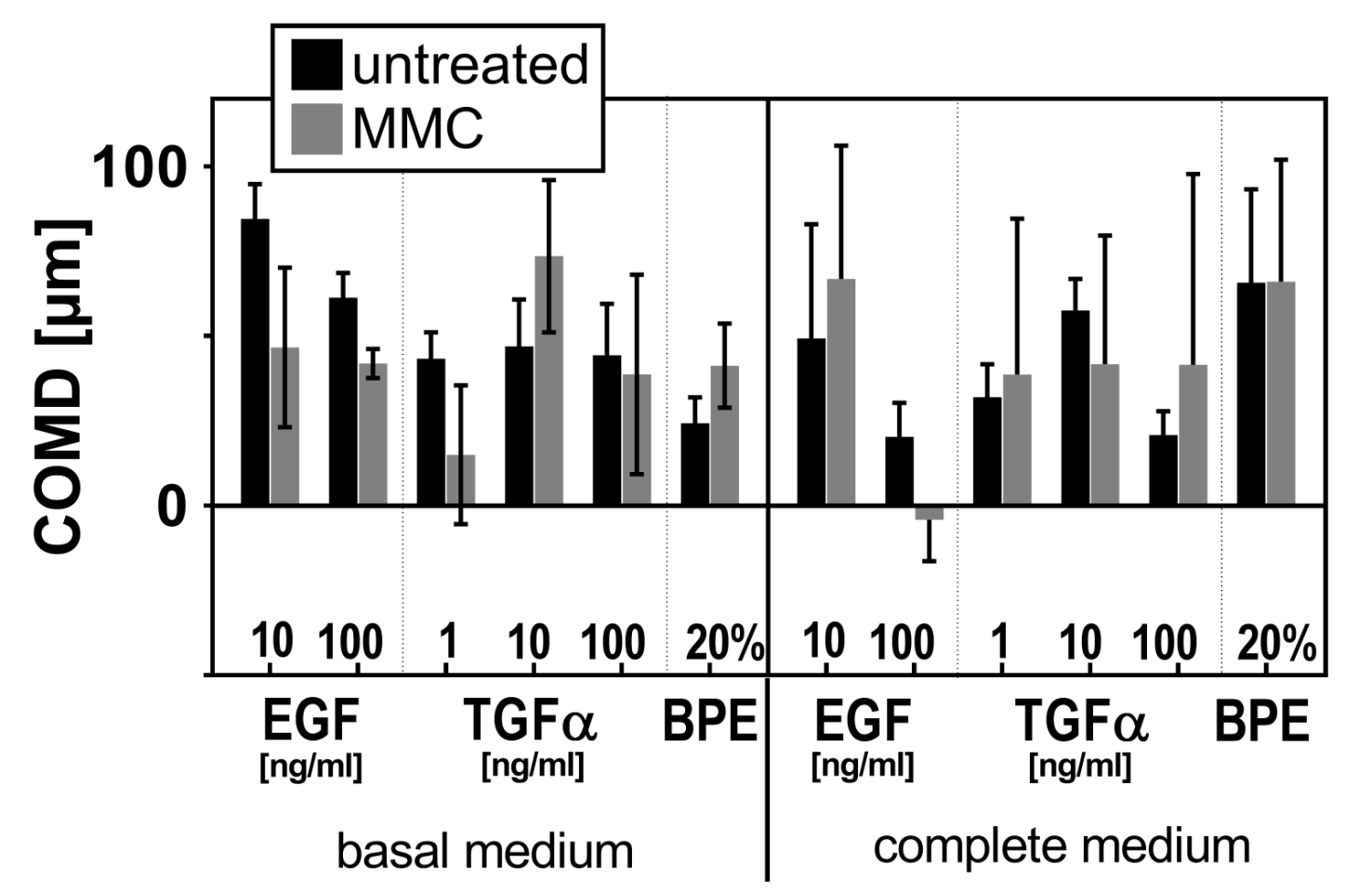

Supplement: S6 Fig — Cell proliferation was inhibited with mitomycin C (MMC) in a control chemotaxis experiment in order to verify that the uneven cell distribution in migration arena is caused by directed migration (true chemotaxis), and is not dependent on cell growth. In order to probe whether increased proliferation of cells in complete medium masked chemotaxis, we used MMC on those samples that gave different results in basal and complete medium (gradients of EGF, BPE). However, no significant differences between MMC-treated and normally proliferating cells were found. Bars show mean COMD ± SEM (4 arenas were analyzed for each condition; each arena contained 150–200 cells). COMD of MMC-treated and untreated cells was compared with multiple t-test; p<0.05). (TIF) [file pone.0219708.s006.tif]

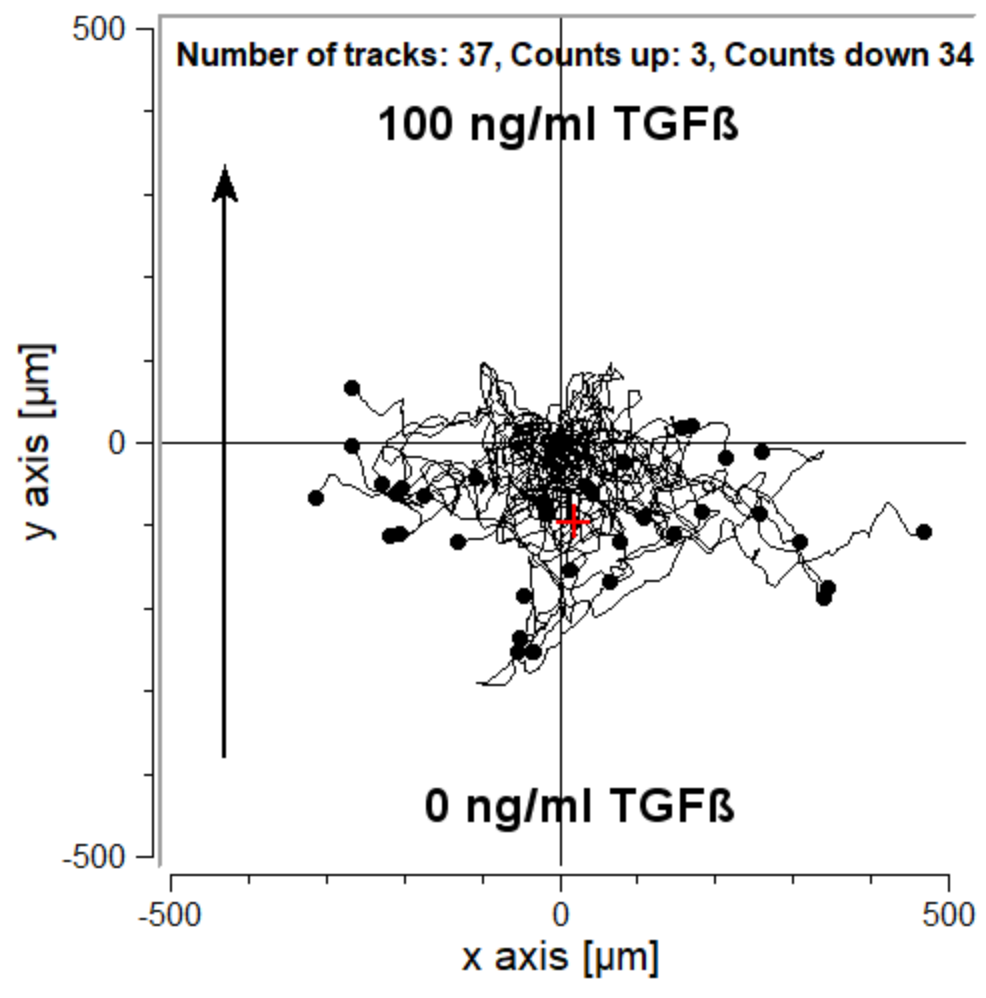

Supplement: S7 Fig — Experiments on nHEK cells (Fig 4) showed a surprising negative chemotaxis effect of a 0–100 ng/ml TGFβ gradient in complete medium. In order to verify that the accumulation of the cells at the distant barrier of the migration arena (in respect to the highest TGFβ concentration) was indeed caused by a chemorepellent effect, we analyzed cell trajectories by manual tracking in the time-lapse sequences acquired during this experiment. The hairplot graph shows the complete trajectories of cells that migrated in the migration arena for 24 hours in the gradient of TGFβ (0–100 ng/ml in complete medium). Cell migration was recorded with time-lapse microscopy with an interval of 10 min, and the trajectories of 35–40 randomly selected cells in each arena were reconstructed by manual tracking. The trajectories were analyzed with the Chemotaxis and migration tool software. Values of chemotactic parameters, such as forward migration indices along the gradient direction (FMIy = -0.09 ± 0.05 [a.u.]; n = 3), or center of mass displacement along the gradient direction (COMy = -73 μm ± 18 μm; n = 3) indicate negative chemotaxis. Shown is a representative result of three independent experiments. The direction of the TGFβ gradient is indicated by the arrow. The end-points of the tracked cells are indicated in the plot by the black dots, and the red cross represents the center of mass displacement. The evaluated raw data were acquired in the same experiment that was shown in Fig 4 and S5 Fig. (TIF) [file pone.0219708.s007.tif]
